# Bilateral Scleritis and Neutrophilic Dermatosis With Cytogenetic Chromosomal Aberrancy Related to Pyoderma Gangrenosum: A Case Report of a 20-Year Follow-Up

**DOI:** 10.7759/cureus.82348

**Published:** 2025-04-16

**Authors:** Toshihiko Matsuo, Takehiro Tanaka, Noboru Asada, Mikako Obika, Ryotaro Omichi, Keiji Iwatsuki

**Affiliations:** 1 Department of Ophthalmology, Graduate School of Interdisciplinary Science and Engineering in Health Systems, Okayama University, and Okayama University Hospital, Okayama, JPN; 2 Department of Pathology, Graduate School of Medicine, Dentistry, and Pharmaceutical Sciences, Okayama University, Okayama, JPN; 3 Department of Hematology and Oncology, Graduate School of Medicine, Dentistry, and Pharmaceutical Sciences, Okayama University, Okayama, JPN; 4 Department of General Internal Medicine, Graduate School of Medicine, Dentistry, and Pharmaceutical Sciences, Okayama University, Okayama, JPN; 5 Department of Otolaryngology, Head and Neck Surgery, Graduate School of Medicine, Dentistry, and Pharmaceutical Sciences, Okayama University, Okayama, JPN; 6 Department of Dermatology, Graduate School of Medicine, Dentistry, and Pharmaceutical Sciences, Okayama University, Okayama, JPN; 7 Department of Dermatology, Fukushima Rosai Hospital, Iwaki, JPN

**Keywords:** corneal infiltration, hypopyon, myelodysplastic syndromes, neutrophilic dermatosis, peripheral keratitis, pyoderma gangrenosum, scleritis, sweet syndrome

## Abstract

Pyoderma gangrenosum is a non-infectious autoimmune disease with skin plaques and ulcers in the entity of neutrophilic dermatosis and may have a background of myelodysplastic syndromes. This study reported a 20-year follow-up of a patient with pyoderma gangrenosum and scleritis who showed chromosomal aberrancy from the initial phase and later in the course developed thrombocythemia. A 51-year-old man presented with widespread indurated erythematous plaques with scaling and pustules on the forehead, bilateral eyelids, and nasal bridge, in addition to nodular scleritis in the left eye and ulcer formation of the plaques in the lower legs. Skin biopsy revealed massive dermal infiltration mainly with neutrophils in the absence of neutrophilic vasculitis. Suspected of myelodysplastic syndromes, bone marrow biopsy was normal, while chromosomal aberrancy, 46, XY, del (20) (q11q13.3), was detected. In the diagnosis of neutrophilic dermatosis, probably of pyoderma gangrenosum, he began to have oral prednisolone 20 mg daily and colchicine 1 mg daily, leading to the subsidence of skin lesions. Four months later, he developed nodular scleritis in the right eye and began to use topical 0.1% betamethasone in both eyes. He was stable with only prednisolone 12.5 mg daily until the age of 55.5 years, when he showed an increase of serum lactate dehydrogenase. The bone marrow aspirate disclosed neither blast cell increase nor atypical cells. The same chromosomal aberrancy was repeatedly detected. One year later, he developed breathing difficulty and underwent tracheostomy. Laryngeal lesion biopsy disclosed squamous cell papilloma with human papillomavirus-6. At 60 years old, he showed marginal corneal infiltration in the left eye, and at 61 years old, hypopyon in the right eye. Platelets tended to increase up to 1000 × 10^3^/µL, and bone marrow examinations were recommended but refused by the patient. At the latest follow-up at 71 years old, he was ambulatory in health and stable with a tracheostomy cannula. In conclusion, pyoderma gangrenosum with scleritis occurred in an undetermined hematological malignancy with chromosomal aberrancy.

## Introduction

Scleritis occurs in isolation or as an ophthalmic complication of systemic diseases such as rheumatoid arthritis [1], antineutrophil cytoplasmic antibody (ANCA)-associated vasculitis [2,3], inflammatory bowel disease [4], polychondritis [5], and so on. Scleritis in association with systemic diseases usually presents bilateral lesions and tends to show high activity with a nodular pattern. It sometimes accompanies marginal corneal infiltration, which is designated peripheral keratitis. Scleritis has also been described in association with neutrophilic dermatosis, including pyoderma gangrenosum [6,7] and Sweet syndrome [8,9,10,11].

Pyoderma gangrenosum is a non-infectious autoimmune disease that is characterized by deep cutaneous ulcers after erosion of papules (or plaques), pustules, and vesicles, often in the anterior part of the lower legs [6,7]. As a similar term but in a different clinical entity, pyoderma is an infectious neutrophilic infiltration of the skin and will lead to bloodstream infection and sometimes result in retinal manifestations in the setting of ophthalmology [12]. Sweet syndrome is called acute febrile neutrophilic dermatosis and presents red, elevated skin plaques often around mucocutaneous junctions such as the eyes, mouth, and genital area [8,9,10,11]. Both pyoderma gangrenosum and Sweet syndrome are designated as neutrophilic dermatosis since their clinical presentations may overlap with each other. Behcet disease, which manifests as uveitis, oral aphthous ulcers, genital ulcers, and skin rashes as erythema nodosum, is also included in the entity of neutrophilic dermatosis. Hematologically, it should be noted that patients with pyoderma gangrenosum [13,14,15] and Sweet syndrome [16] may have a background of myelodysplastic syndromes. Inflammatory bowel disease [17] and Behcet disease [18,19] have also been reported to have a background of myelodysplastic syndromes. In this report, we describe the long-term follow-up for two decades of a patient who developed bilateral nodular scleritis and pyoderma gangrenosum in the background of cytogenetic chromosomal aberrancy.

## Case presentation

**Figure 1:**
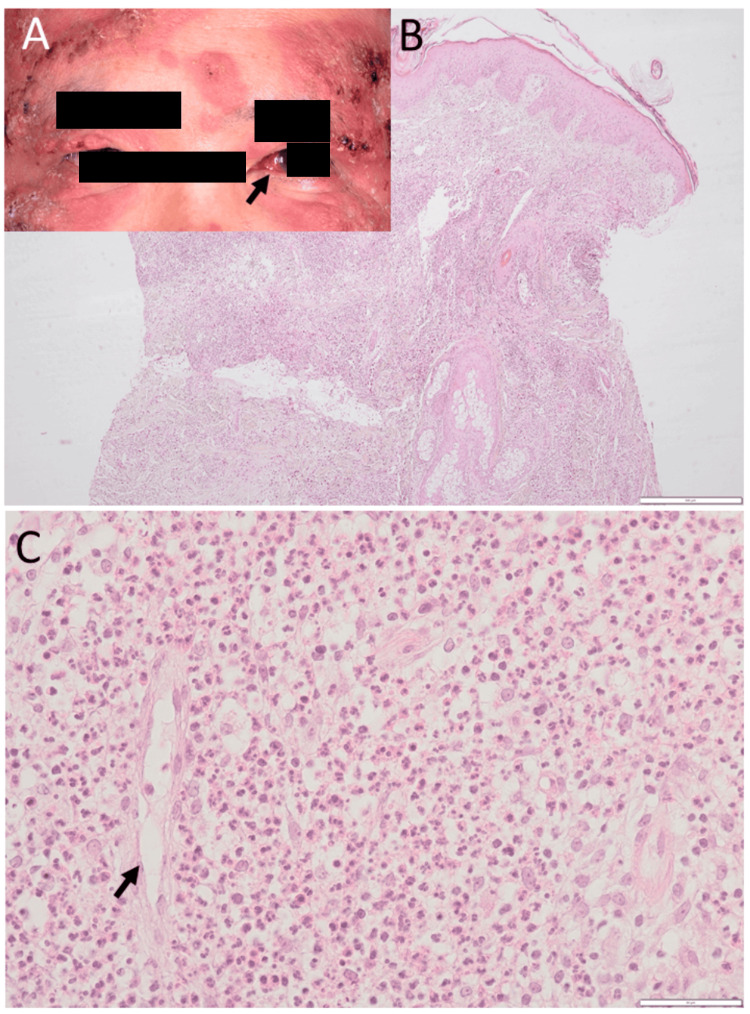
Skin lesions, scleritis, and skin biopsy at the age of 51 years Elevated erythematous plaques (A) with pustules on the forehead skin and bilateral eyelids, most likely Sweet syndrome, as well as scleritis (arrow) on the left side at the initial visit at the age of 51 years. Skin biopsy of the forehead, showing massive dermal infiltration with neutrophils under keratinized stratified epithelium at low magnification (B). Predominant neutrophils with a small number of eosinophils and monocytes at high magnification (C). Note no vascular infiltration (arrow in C), indicative of pyoderma gangrenosum. Scale bar = 500 µm in B, 50 µm in C.

A 51-year-old man noticed a three-week rapid exacerbation of skin rashes in the face and chest, which had been smoldering for a year. At the initial visit to a dermatologist, he showed widespread elevated and indurated erythematous plaques with scaling and pustules on the entire forehead (Figure 1A), bilateral upper and lower eyelids (Figure 1A), and nasal bridge, suggestive of Sweet syndrome. The isolated erythematous plaques were additionally found in the bilateral chest, palms, and backs of bilateral hands, and the anterior part of bilateral upper and lower legs with ulcer formation, suggestive of pyoderma gangrenosum. He also showed nodular scleritis on the nasal side of the left eye (Figure 1A). The best-corrected visual acuity in decimals was 1.2 in both eyes. Ophthalmic examinations revealed no inflammation in the anterior chamber and fundus of both eyes. He had no fever or general fatigue. Physical examinations were normal. In history, he underwent an appendectomy at the age of 18 years. He quit smoking at the age of 45 years after he had smoked 20 cigarettes daily for 25 years. He was an occasional drinker until the initial visit, but he quit drinking alcohol afterward. Family history revealed lung cancer in his father and maternal grandfather, lymphoma in his mother, and pancreatic cancer in his mother’s brother and maternal cousin. At the initial visit, complete blood cell counts and blood chemistry tests were in the normal range (Table 1). Immunoglobulin G and M (IgG and IgM) were normal at 1491 mg/dL and 243.1 mg/dL, respectively, while immunoglobulin A (IgA) was elevated at 565.9 mg/dL. C-reactive protein was high at 4.0 mg/dL, and the erythrocyte sedimentation rate was high at 115 mm/one hour. Ferritin was normal at 210.2 ng/mL, and antinuclear antibody and rheumatoid factor were negative. Serological tests for syphilis as well as screening tests for hepatitis B virus antigen, hepatitis C virus antibody, and human immunodeficiency virus antigen/antibody were all negative.

**Table 1:** Blood examinations at the age of 51 years in the initial visit, at the age of 55.5 years, and at the age of 71 years in the latest visit Normal ranges at the in-house laboratory were at the time he was 51 years old. All values are reported in standard units. LD: lactate dehydrogenase; AST: aspartate aminotransferase; ALT: alanine aminotransferase; γ-GT: γ-glutamyl transferase; eGFR: estimated glomerular filtration rate; CRP: C-reactive protein.

Test	Normal range	At 51 years	At 55.5 years	At 71 years
Red blood cells (x 10^6^/µL)	4.00-5.60	4.12	4.12	4.16
Platelets (x 10^3^/µL)	150-400	250	352	1040
White blood cells (x 10^3^/µL)	3.0-9.4	8.4	7.46	5.32
Segmented neutrophils (%)	29.0-70.0	48.0	74.2	78.0
Stab neutrophils (%)	0.0-13.0	18.0	5.5	5.5
Lymphocytes (%)	20.0-52.0	13.0	16.2	10.0
Monocytes (%)	0.0-13.0	11.0	6.2	5.0
Eosinophils (%)	0.0-11.0	9.0	3.2	0.0
Basophils (%)	0.0-2.0	0.0	0.2	0.5
Atypical cells (%)	0.0	1.0	0.0	0.5
Hemoglobin (g/dL)	13.5-17.5	13.4	12.0	12.7
Hematocrit (%)	40.0-52.0	39.7	36.6	42.1
Total protein (g/dL)	6.50-8.00	7.67	7.0	5.7
Albumin (g/dL)	3.90-4.90	3.76	3.0	3.1
LD (U/L)	120-240	263	608	1196
AST (U/L)	11-32	19	26	23
ALT (U/L)	6-39	16	25	24
γ-GT (U/L)	3-40	23	29	35
Total bilirubin (mg/dL)	0.33-1.28	0.39	0.29	0.31
Urea nitrogen (mg/dL)	8.1-22.0	19.4	10.2	19.0
Creatinine (mg/dL)	0.44-1.04	1.00	0.91	1.36
eGFR (mL/min/1.73 m^2^)	60 or greater	n.d.	64.1	40.8
Uric acid (mg/dL)	3.9-6.9	6.9	6.9	6.2
Total cholesterol (mg/dL)	130-220	146	103	n.d.
CRP (mg/dL)	0.0-0.3	4.0	7.05	0.18

**Figure 2:**
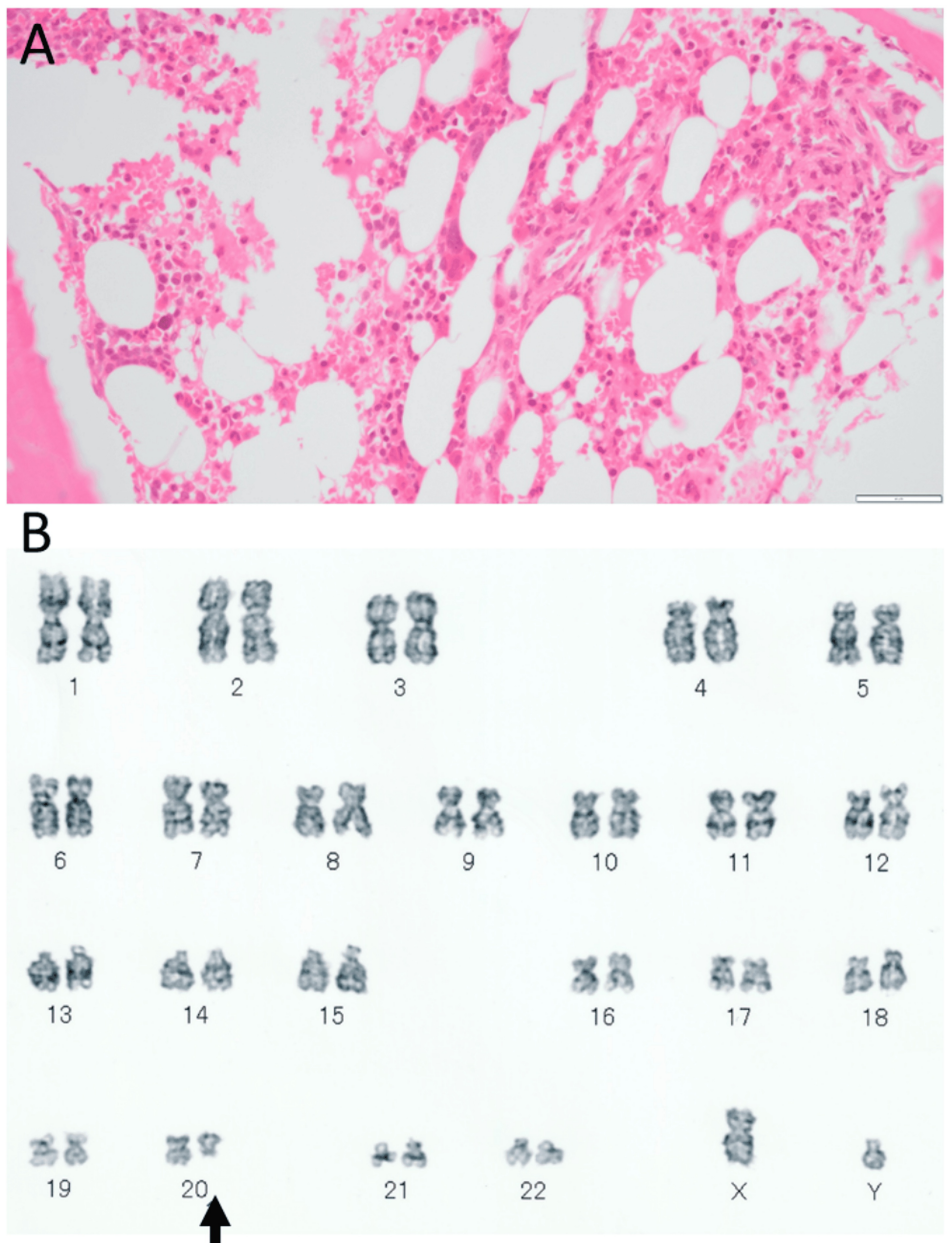
Bone marrow biopsy and karyotyping at the age of 51 years Normally appearing bone marrow in hematoxylin-eosin stain (A) by biopsy two weeks from the initial visit. Chromosomal aberrancy, 46, XY, del (20) (q11q13.3) detected by the G-band method (arrow, B) in 20 out of 20 tested cells of the bone marrow aspirate. Scale bar = 50 µm.

**Figure 3:**
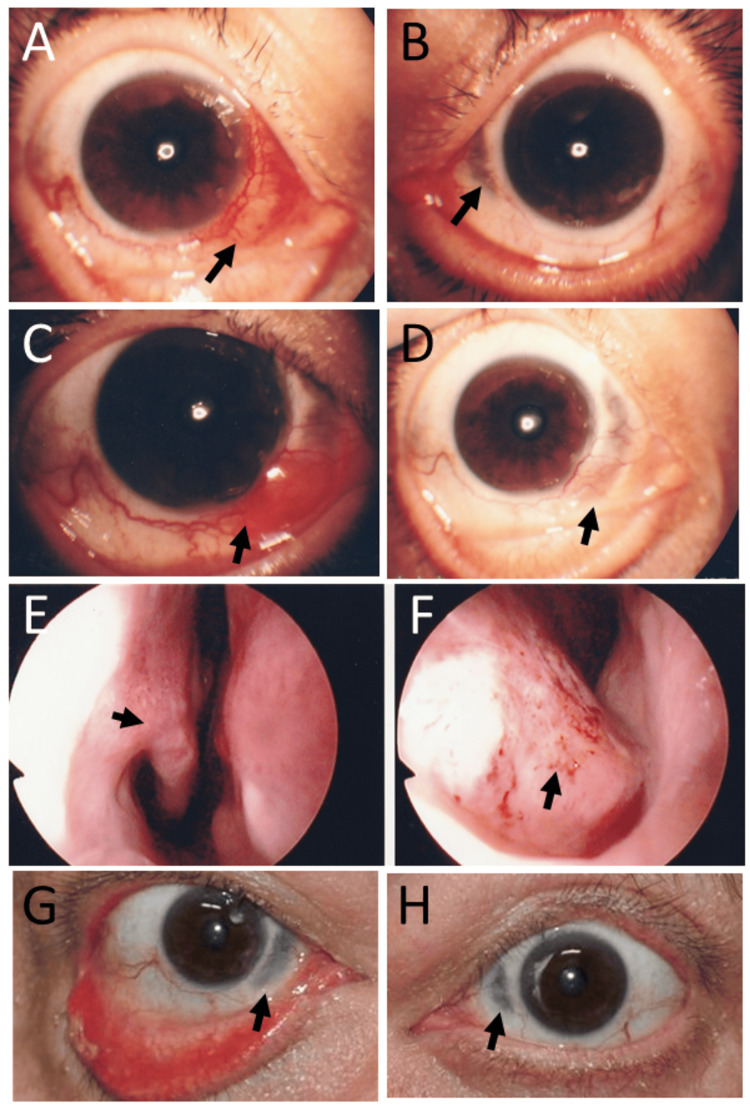
Time course of scleritis and nasal granuloma at the age of 51 years and subsided scleritis at the age of 58 years Four months later from the initial visit, nodular scleritis (arrow, A) on the nasal side in the right eye and thin sclera (arrow, B) in the left eye to see through choroidal pigmentation after scleritis has subsided. Six months after the initial visit, nodular scleritis (arrow, C) in the right eye became more active but subsided in three weeks (arrow, D). Nasal inferior turbinate granuloma (arrow, E) on the right side and nasal septum granuloma (arrow, F) on the left side by nasal mirror 10 months from the initial visit. At the age of 58 years, seven years later from the initial visit, thin sclera with no active scleritis in both eyes (arrows, G in the right eye and H in the left eye) with lower eyelid ectropion on the right side.

Skin biopsy of the forehead lesion revealed massive dermal infiltration mainly with neutrophils (Figure 1B), which were admixed with a small number of eosinophils and monocytes (Figure 1C). Microorganisms were not detected by the Giemsa and Gram stains. Neutrophilic vasculitis was absent (Figure 1C), supporting the diagnosis of pyoderma gangrenosum. Due to a suspicion of myelodysplastic syndromes, he underwent a bone marrow biopsy, but it revealed a normal-appearing bone marrow (Figure 2A). Bone marrow aspiration also showed differential cell counts within normal limits and disclosed no anomalous cells. In the time course, chromosomal aberrancy, 46, XY, del (20) (q11q13.3), was detected in 20 of 20 tested cells of the bone marrow aspirate and in two of two tested cells derived from the peripheral blood by a G-band method (Figure 2B). Plasma granulocyte-colony stimulating factor (G-CSF) was at 59 pg/mL, slightly above the upper limit of the normal range. With the diagnosis of neutrophilic dermatosis, probably of pyoderma gangrenosum, he began to have oral prednisolone 20 mg daily and colchicine 1 mg daily, leading to the subsidence of skin lesions. Topical 0.1% betamethasone eye drops four times daily were given to the left eye with nodular scleritis. Two months after the initial visit, oral prednisolone was tapered to 15 mg daily, and colchicine was discontinued. Four months after the initial visit, he developed nodular scleritis on the nasal side in the right eye (Figure 3A) at a dose of prednisolone of 12.5 mg daily while scleritis in the left eye had subsided (Figure 3B). He began to use 0.1% betamethasone eye drops four times daily in both eyes. Six months later, from the initial visit, when oral prednisolone was tapered to 7.5 mg daily, nodular scleritis temporarily exacerbated (Figure 3C) but subsided in three weeks (Figure 3D). At this point, oral prednisolone was lowered to 5 mg daily. Ten months after the initial visit, he had hoarseness and was referred to an otolaryngologist to have nasal inferior turbinate granuloma on the right side (Figure 3E) and nasal septum granuloma on the left side (Figure 3F) as well as suspected laryngeal granuloma. Oral prednisolone was thus increased to 12.5 mg daily and was maintained at this dose afterward.

**Figure 4:**
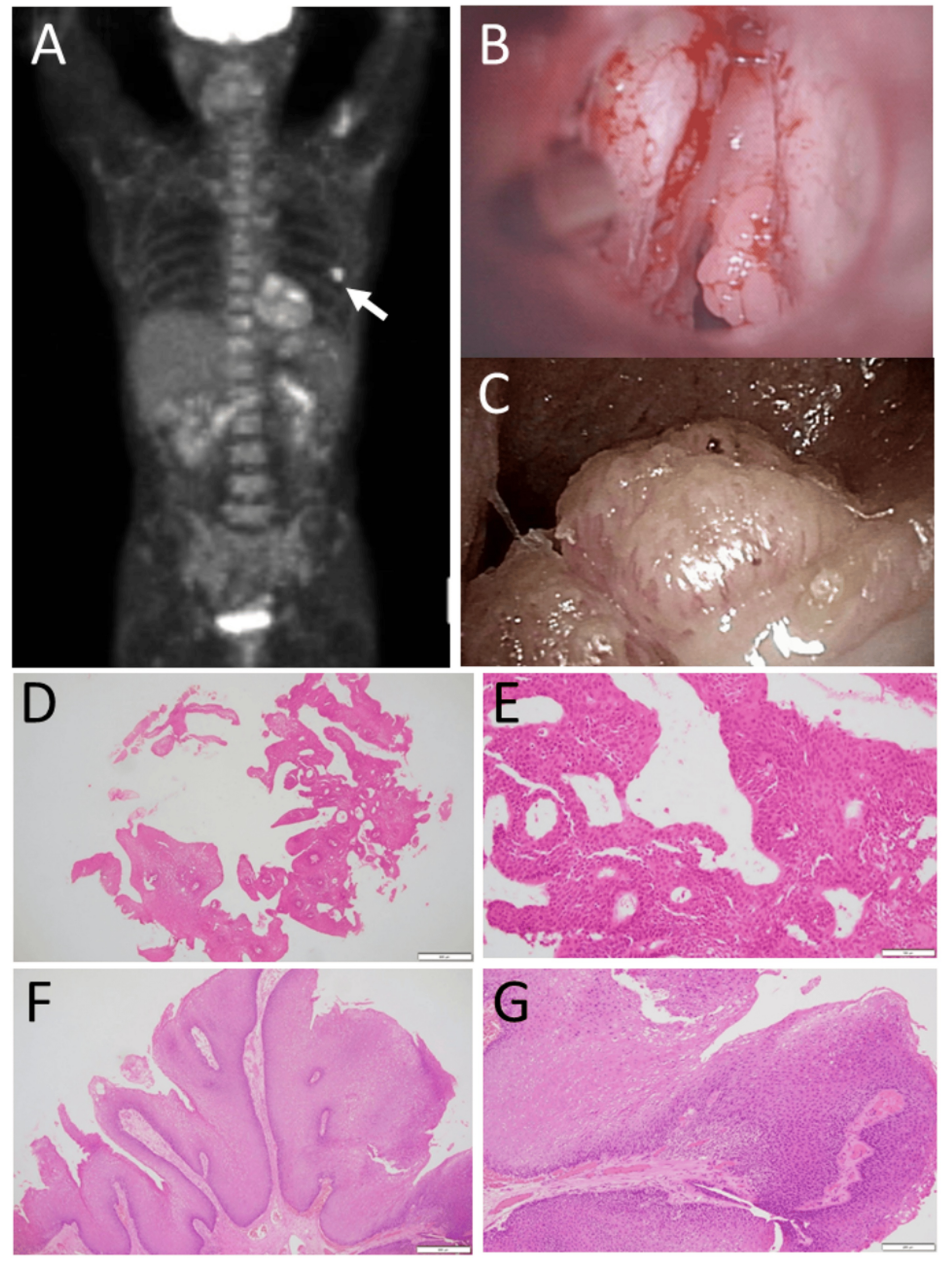
Positron emission tomography at the age of 55 years, vocal cord papillomatous lesion at the age of 56 years, and laryngeal papilloma at the age of 67 years Positron emission tomography at the age of 55 years, 4.5 years later from the initial visit, showed high uptake in the back skin nodule on the left side (arrow, A) and somewhat increased uptake in the bone marrow. Vocal cord papillomatous lesions and left vocal cord edema (B) at the age of 56 years, 5.5 years later from the initial visit when he developed breathing difficulty. Biopsy of the lesion, showing squamous cell papilloma (D, E). Enlarged laryngeal papilloma (C) before resection at the age of 67 years, 16 years later from the initial visit. Laryngoscopic resection of the lesion, showing squamous cell papilloma (F, G). Hematoxylin-eosin stain. Scale bar = 500 µm in D and F, 100 µm in E, 200 µm in G.

He was stable until the age of 55.5 years, 4.5 years later from the initial visit, when he showed an elevated level of lactate dehydrogenase (LD) at 608 U/L (Table 1). The differentials of LD isozymes were LD1 at 22.8%, LD2 at 43.7%, LD3 at 25.2%, LD4 at 5.3%, and LD5 at 3.0%, suggestive of a lymphoma and leukemia pattern. He had a whole-body fluorodeoxyglucose (FDG)-PET scan, which showed a lot of activity in a skin nodule on the left side of his back and a bit more activity in the bone marrow (Figure 4A). The bone marrow aspirate at that time disclosed neither blast cell increase nor atypical cells. The same chromosomal aberrancy, 46, XY, del (20) (q11q13.3), was repeatedly detected in 16 of 20 tested cells of the bone marrow aspirate and one of the one tested cell derived from the peripheral blood by the G-band method, while the remaining four cells of the bone marrow aspirate showed the normal karyotype. One year later, at the age of 56.5 years, he was admitted to an emergency room because of breathing difficulty. He underwent tracheostomy under local anesthesia, and the laryngeal lesion (Figure 4B) was diagnosed pathologically by biopsy as squamous cell papilloma (Figures 4D, 4E). Human papillomavirus-6 was detected by polymerase chain reaction in the lesion. In this period of six years, scleritis in both eyes remained inactive (Figures 3G, 3H).

**Figure 5:**
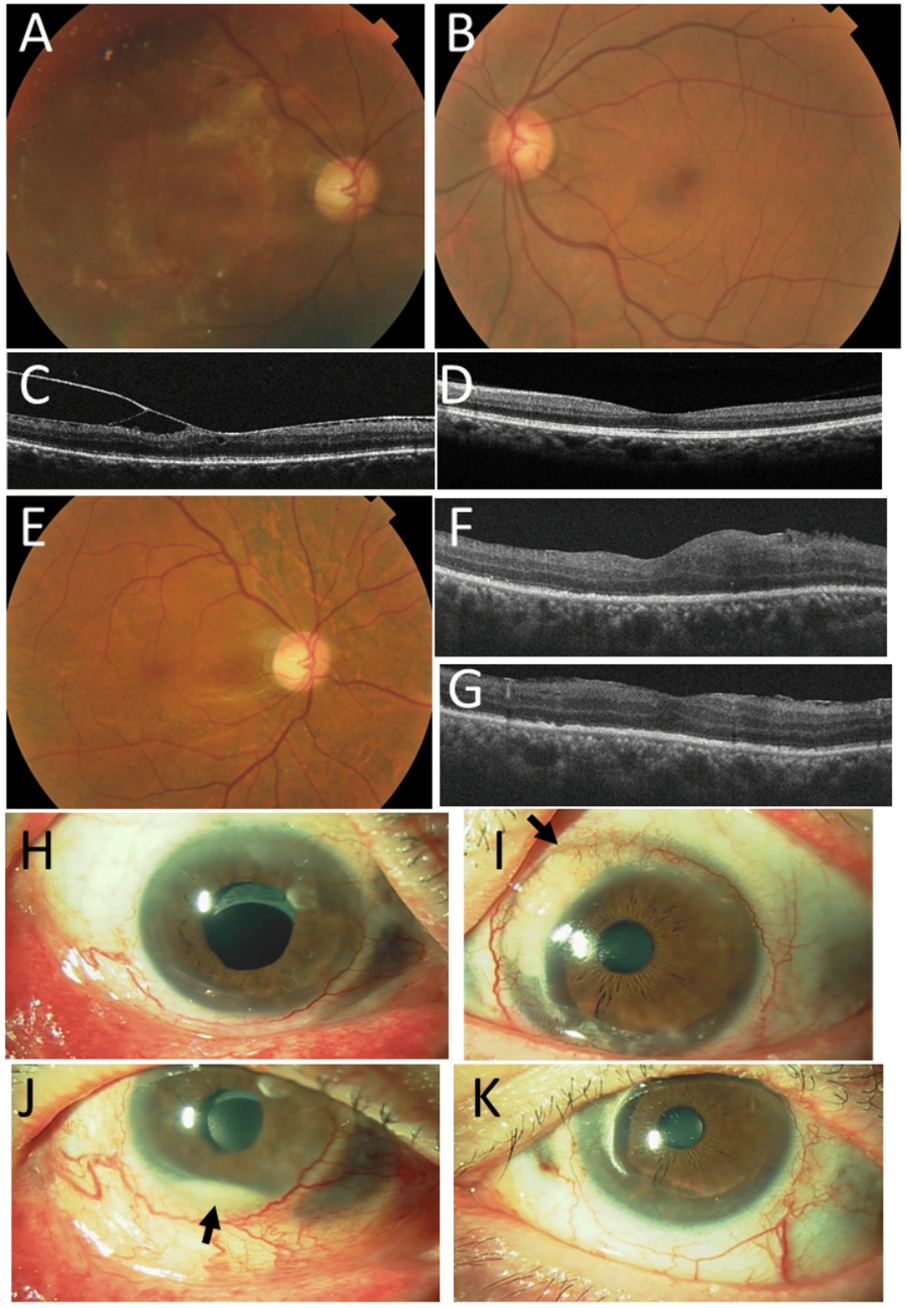
Fundus photographs, optical coherence tomography, and slit-lamp photographs from the age of 58 years to 61 years Fundus photographs (A, B) and optical coherence tomography (C, D) in the horizontal section show secondary epiretinal membrane formation in the right eye (A, C) and a normal macula in the left eye (B, D) at the age of 58 years, 7.5 years later from the initial visit. Fundus photograph (E) and optical coherence tomography in the horizontal section (F) and vertical section (G) show a normally appearing retina in the right eye one month after vitrectomy. No active scleritis in the right eye (H) and marginal corneal active infiltration on the nasal to upper side (arrow, I) in the left eye at the age of 60 years, nine years later from the initial visit. Hypopyon in the right eye (arrow, J) and peripheral corneal opacity sequel to marginal keratitis in the left eye (K) at the age of 61 years.

At the age of 58 years, 7.5 years later from the initial visit, he underwent cataract surgery with intraocular lens implantation for corticosteroid-induced cataract in the right eye. Three months later, he underwent vitrectomy to peel off the epiretinal membrane with vitreomacular traction in the right eye, which would probably be caused by inflammation (Figures 5A-5G). At the age of 60 years, he showed marginal corneal infiltration on the nasal to upper side in the left eye (Figures 5H, 5I), and he took temporary additional oral prednisolone 10 mg daily in the baseline administration of 12.5 mg. At the age of 61 years, he showed hypopyon in the right eye (Figures 5J, 5K), and temporary oral prednisolone 5 mg daily was added to the baseline dose of 12.5 mg.

At the age of 64.5 years, he underwent laryngoscopic laser ablation four times in half a year. At the age of 66 years, he had a vaccination for human papillomavirus (human papillomavirus quadrivalent types 6, 11, 16, and 18 vaccines, recombinant injection, suspension, Merck Sharp and Dohme, Inc., Rahway, NJ, USA). At the age of 67.5 years, he underwent laryngoscopic extirpation of papilloma in the larynx (Figures 4C, 4F, 4G). At the age of 70 years, he had a headache of three-week duration and underwent surgery for chronic subdural hematoma with cerebrospinal fluid leakage, although he did not have an episode of blunt head trauma. Platelets were 1230 x 10^3^/µL at that time. Throughout the course after the last bone marrow examination at the age of 55.5 years, platelets tended to increase up to 1000 x 10^3^/µL, and bone marrow examinations were recommended but refused by the patient. Suspicious of essential thrombocythemia, genomic DNA analysis of the peripheral blood sample showed no pathogenic mutations regarding *Janus kinase-2* (*JAK2*) V617F, *JAK2* exon 12,* calreticulin* (*CALR*) exon 9, and myeloproliferative leukemia virus oncogene (*MPL*) W515L/K. Pathogenic variants in the breakpoint cluster region-*Abelson-1* (*BCR::ABL1 gene*) were also absent.

**Figure 6:**
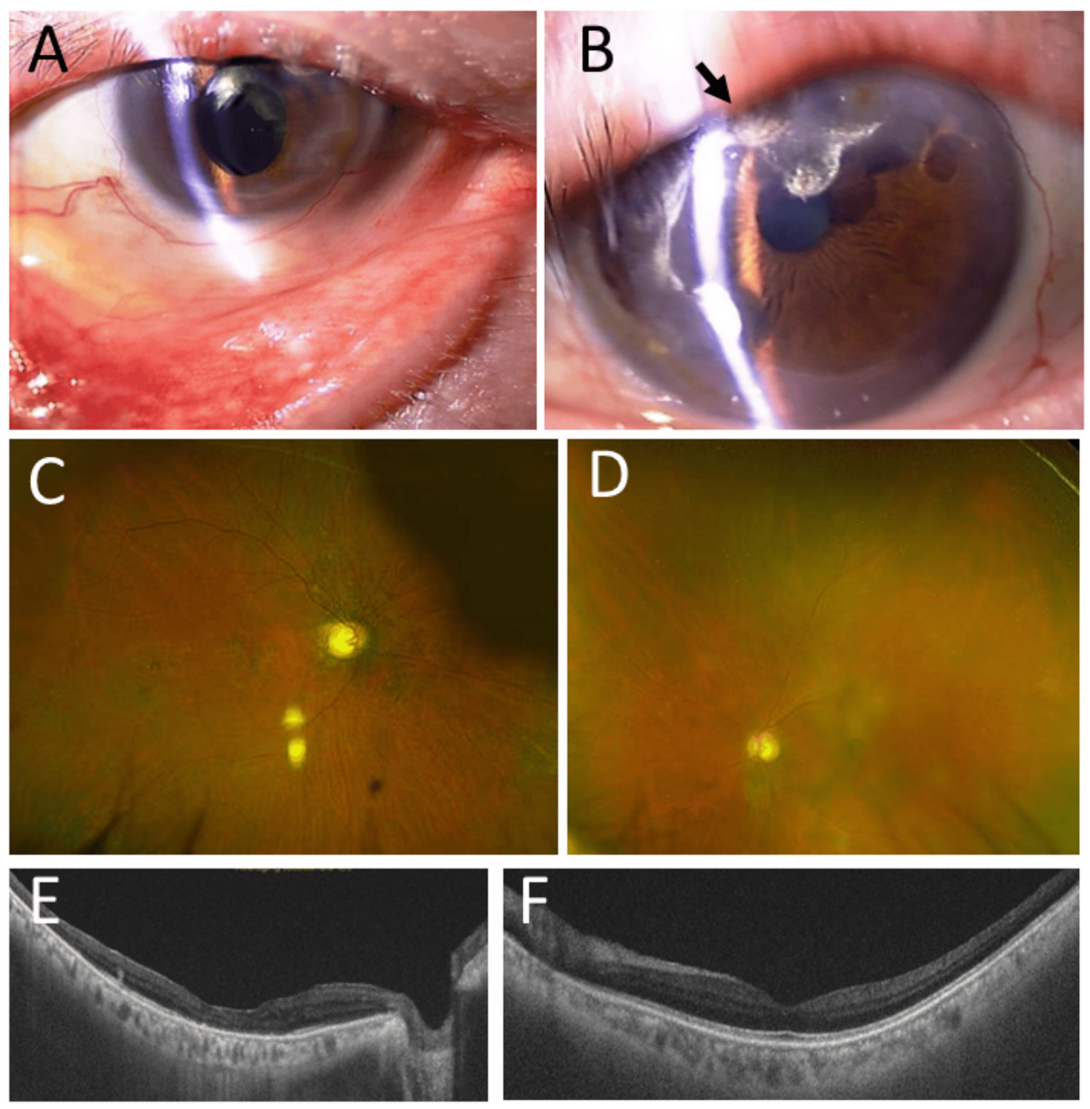
Slit-lamp photographs, wide-field fundus photographs, and optical coherence tomography at the age of 71 years At the age of 71 years, 20 years later from the initial visit, mild conjunctival invasion into the cornea on the nasal side in the right eye with intraocular lens implantation and lower eyelid ectropion (A); marked conjunctival invasion into the cornea (arrow) on the upper to nasal side in the left eye with mild cataract (B). Wide-field fundus photographs and horizontal sections of optical coherence tomography show optic disc atrophy (C) and thinned macular retina (E) in the right eye, normal optic disc color (D), and normal macula in the left eye (F).

At the latest follow-up at the age of 71 years, he was ambulatory in health and stable with a tracheostomy cannula. He had splenomegaly. He continued oral prednisolone 12.5 mg daily and had linagliptin 5 mg, insulin degludec once daily, and insulin lispro before each meal for corticosteroid-induced diabetes mellitus, and febuxostat 10 mg for hyperuricemia. Complete blood cell counts and blood chemistry tests (Table 1) showed a high number of platelets at 1040 x 10^3^/µL and a high level of LD at 1196 U/L. Creatinine was slightly elevated to 1.36 mg/dL, and the estimated glomerular filtration rate (eGFR) was reduced to 40.8 mL/min/1.73 m². Hemoglobin A1c was 7.7%. The scleritis in both eyes remained inactive with 0.1% betamethasone once daily (Figures 6A, 6B). The right eye showed optic disc atrophy (Figure 6C) and macular retinal thinning (Figure 6E), while the left eye maintained normal optic disc color (Figure 6D) and macular structure (Figure 6F). The best-corrected visual acuity in decimals was 0.06 in both eyes, due to corneal astigmatic deformation sequel to scleritis and marginal keratitis.

## Discussion

The present patient showed skin lesions that showed clinical characteristics in a mixture of pyoderma gangrenosum and Sweet syndrome. The facial manifestations were more consistent with the diagnosis of Sweet syndrome, while ulcer formation in granulomatous skin lesions in the legs was consistent with the diagnosis of pyoderma gangrenosum. Pathological examinations of skin lesion biopsy at the initial visit disclosed massive dermal infiltration with neutrophils in a mixture of a small number of eosinophils and monocytes. The absence of perivascular and intravascular infiltration with neutrophils is a hallmark of pyoderma gangrenosum and can be used pathologically to exclude Sweet syndrome. Infectious causes for neutrophilic infiltration in the skin were also excluded by the absence of bacteria on Gram stain.

Since pyoderma gangrenosum is known to be associated with rheumatoid arthritis, inflammatory bowel disease [17], and myelodysplastic syndromes [13,14], the bone marrow was examined, and the peripheral blood was analyzed for karyotype cytogenetic tests by a G-band method to search for chromosomal aberrancy [20]. The bone marrow had normal morphology with no increase in blast cells. The G-band showed del (20) (q11q13.3) as a chromosomal aberrancy. In general, the diagnosis of myelodysplastic syndromes is based on three pillars: cytopenia, morphologic dysplasia, and genetic evidence of clonality [20]. The present patient did not show cytopenia in any of the three hematopoietic lineages, and also did not show blast cell increase in the bone marrow and peripheral blood. He did show the same cytogenetic abnormal karyotype in the bone marrow and peripheral blood on repeated occasions. Therefore, the diagnosis at the initial visit would be subclinical myelodysplastic syndrome with del (20) (q11q13.3). In the face of increasing levels of serum LD, he underwent a second bone marrow examination 4.5 years later from the initial visit, but did not show abnormal findings, except for the same chromosomal aberrancy. PET/CT at that time showed a non-specific increase of uptake in the bone marrow, suggestive of bone marrow diseases [21]. 

In two decades of the clinical course, the present patient showed a gradual increase in platelets, which was suspected of essential thrombocythemia in the late course of follow-up with splenomegaly. He also did not show any pathogenic variants in genes that would be suggestive of essential thrombocythemia. The exact diagnosis of essential thrombocythemia could not be made since he refused to undergo bone marrow biopsy and aspiration. Under the circumstances, the large number of platelets in the peripheral blood is at least against the diagnosis of myelodysplastic syndromes, which have been made at the initial visit. Chronic subdural hematoma without any preceding episode of head trauma in this patient might be attributed to the abnormal state of coagulation caused by thrombocythemia [22].

Hematological diagnosis in the present patient is challenging in 20 years of follow-up: he did not show cytopenia in any lineage throughout the course, while he developed a gradual increase of platelets, indicative of thrombocythemia, together with splenomegaly. Karyotypic analysis of bone marrow aspirates and peripheral blood at the initial visit and four to five years later on the second examination repeatedly showed the same chromosomal aberrancy, del (20) (q11q13.3). It should be noted that 20% of the tested cells of the bone marrow aspirate on the second examination showed the normal karyotype, indicative of somatic mosaicism in the bone marrow population. The chromosomal aberrancy, del (20q12), has been known to show refractory thrombocytopenia with a low risk for progression to acute myelogenous leukemia [23], which is against the thrombocythemia in the present patient. Myelodysplastic syndromes and essential thrombocythemia as myeloproliferative neoplasms have been reported to develop in the course of the same patient [24,25], and the entity of myelodysplastic syndrome/myeloproliferative neoplasm overlap syndromes has been recognized [26]. Indeed, somewhat similar to the present patient, myelodysplastic/myeloproliferative neoplasm with ring sideroblasts and thrombocytosis was reported to develop in an old patient with an abnormal karyotype of 46, XX, del (20) (q1?) and *JAK2* V617F mutation but not *CALR* type 1 or 2 mutations [27]. From different points of view, neutrophilic dermatoses with different entities such as pyoderma gangrenosum, Sweet syndrome, and vacuoles, E1 enzyme, X-linked, autoinflammatory, somatic (VEXAS) syndrome, in addition to Behcet disease, are characterized as a kind of autoinflammatory disease to overdrive neutrophils in the background of hematological abnormalities [28]. VEXAS syndrome shows vacuoles in myeloid and erythroid lineage cells in the bone marrow and thrombocytopenia, which is against thrombocythemia in the present patient [29].

The patient’s ophthalmic involvement initially manifested as bilateral nodular scleritis. However, during maintenance therapy with 12.5 mg daily prednisolone, which was sufficient to suppress skin lesions, episodes of ophthalmic relapse occurred, presenting either peripheral keratitis or hypopyon. On the occasions of ophthalmic relapse, an additional dose of prednisolone 5 mg or 10 mg daily was prescribed by an ophthalmologist in addition to the basic dose of 12.5 mg daily prescribed by a dermatologist. The additional dose of prednisolone was determined in the dialogue with the patient, and the duration of the additional dose was set at a limited period of time, for instance, two weeks or a month until the subsidence of ophthalmic relapse.

The clinical course in the present patient was complicated by laryngeal lesions, which caused breathing difficulty. At the initial visit, the nasal and laryngeal lesions were thought to be granuloma, which would be related to pyoderma gangrenosum. At the presentation of the emergency, 5.5 years later from the initial visit, the laryngeal lesion was pathologically examined to prove to be a squamous cell papilloma caused by human papillomavirus-6. Repeated episodes of resection showed consistently the same pathological diagnosis. In contrast, the nasal lesions at the initial visit appeared to be granulomatous lesions caused by pyoderma gangrenosum since the nasal lesions subsided in response to the increased dose of prednisolone.

## Conclusions

The 20-year follow-up was made in a patient with pyoderma gangrenosum who also showed ophthalmic manifestations of scleritis, peripheral keratitis, and hypopyon. In the long-term course, he was diagnosed with subclinical myelodysplastic syndromes based on cytogenetic karyotype aberrancy but was later complicated with the marked increase of platelets in the presence of splenomegaly, suspected of essential thrombocythemia. The hematological manifestations in the background of the same chromosomal aberrancy may change the face in the long course of observation. Pyoderma gangrenosum and Sweet syndrome in the entity of neutrophilic dermatosis may have overlapping skin manifestations, and a skin biopsy will present a hint for differential diagnosis. These two diseases may share a common feature of having hematological abnormalities as myelodysplastic syndromes, and thus should be examined hematologically.
